# Inhibitory PAS domain protein is a substrate of PINK1 and Parkin and mediates cell death in a Parkinson's disease model

**DOI:** 10.1038/cddis.2015.243

**Published:** 2015-09-17

**Authors:** S Kasai, S Torii, A Kakita, K Sogawa

**Affiliations:** 1 Department of Biomolecular Sciences, Graduate School of Life Sciences, Tohoku University, Sendai, Japan; 2 Department of Pathology, Brain Research Institute, University of Niigata, Niigata, Japan

Inhibitory PAS domain protein (IPAS) was first reported by Lorenz Poellinger and his co-workers^1^ at Karolinska Institutet in 2001 as a potent negative transcriptional regulator of hypoxia-inducible factor-1 (HIF-1), a master regulator of mammalian oxygen homeostasis. IPAS directly binds to the *α*- subunit of HIF-1 and abrogates its DNA binding. They also found that IPAS is mainly expressed in neural tissues, including cerebellar Purkinje cells. Soon after the discovery, it was found that IPAS is a splice variant of HIF-3*α*,^2^ which has numerous splicing variants, and most of which have inhibitory activity against HIF-1 with a different mode of action from IPAS. In 2011, we reported a novel function of IPAS as a proapoptotic factor acting on mitochondria and inducing apoptosis through direct binding to prosurvival proteins, including Bcl-x_L_, Bcl-w, and Mcl-1.^3^ The physical interaction between IPAS and prosurvival factors resulted in liberation and activation of Bax, leading to the release of cytochrome *c* from the mitochondria and activation of caspase-3.

We then investigated physiological and pathological processes in which proapoptotic activity of IPAS is involved. Considering that IPAS is expressed in neural tissues and that proapoptotic activity of IPAS is activated by oxidative stress via the classical NF-*κ*B pathway in pheochromocytoma PC12 cells,^4^ we focused on the neuronal cell death in Parkinson's disease (PD). It is widely recognized that oxidative stress has a key role in the etiology of PD,^5^ and activation of NF-*κ*B was previously reported in animal models of PD.^6^ A considerable body of evidence, as described below, from *in vitro* analysis of interaction between IPAS and PTEN-induced putative kinase 1 (PINK1)-Parkin, analysis of neurotoxin-based PD models using IPAS^−/−^ mice, and expression analysis of IPAS in post-mortem brains of patients with PD, clearly demonstrated that IPAS is an important protein in neurodegeneration in PD.^7^

Mutation in the Parkin gene (*PARK2*) or PINK1 gene (*PARK6*) causes autosomal recessive juvenile PD.^8^ Recently, it was elucidated that upstream PINK1 phosphorylates Parkin to regulate mitophagy for quality control of mitochondria.^9^ Thus, we investigated whether IPAS is a substrate of PINK1 and Parkin, and positive results were obtained. When cultured cells (SH-SY5Y and HeLa cells) were treated with carbonyl cyanide *m*-chlorophenyl hydrazone (CCCP), a reagent for mitochondrial depolarization that causes activation of the PINK1-Parkin pathway, overexpressed IPAS was phosphorylated by PINK1 at several sites including Thr12 as shown in Figure 1. Substitution of Thr12 with Ala led to a total loss of ubiquitination of IPAS by Parkin. Ubiquitin chains of IPAS were predominantly K48 linkages, which are known as linkages leading to proteasomal degradation of the target proteins. Ubiquitination sites were investigated by replacing Lys residues with Arg residues. A lysine cluster present in the N-terminal half (aa 167–169) was found to be a main target of ubiquitination. Turnover of IPAS on the mitochondria was strongly increased by CCCP treatment. Pathogenic missense mutations of Parkin present in UBL, RING0, RING1 and IBR and RING2 domains exhibited a little or no ubiquitination activity toward IPAS except for R42P in the UBL domain, which catalyzed enhanced ubiquitination of IPAS for unknown reasons. Overexpression of cerulean-IPAS induced apoptosis in transfected SH-SY5Y cells. Treatment of cells with CCCP nearly perfectly abolished the apoptosis, and notably the inhibition of apoptosis was reversed by concomitant knockdown of Parkin. Taken together, these results strongly suggest that decreased activity of PINK1-Parkin stabilizes IPAS and increases its protein levels on the mitochondria, leading to apoptosis in cells.

1-Methyl-4-phenyl-1,2,3,6-tetrahydropyridine (MPTP) is the most widely used toxin to produce mammalian models of PD. To clarify the involvement of IPAS in the degeneration of dopaminergic neurons in substantia nigra pars compacta (SNpc), induction of IPAS was first of all examined following MPTP administration. IPAS mRNA was rapidly and strongly induced 2 to 4 h after administration of MPTP (see Figure 1) and its enhanced expression returned to the basal level within 12 h. Immunohistochemical analysis showed that induced IPAS protein was expressed mainly in the cytoplasm of dopaminergic neurons in the SNpc. Next, susceptibility to MPTP in male IPAS^−/−^ mice, which lack the IPAS-specific last exon, was investigated. The mutant mice were fully viable, fertile, apparently normal in appearance and behavior, and expression of other HIF-3*α* splicing variants appeared not to be disturbed. However, acute administration of MPTP to IPAS^−/−^ mice displayed only a modest decrease in the number of tyrosine hydroxylase (TH)-positive neurons, whereas the treatment significantly reduced the number of TH-positive neurons in wild-type littermates. These results demonstrate that IPAS is involved in the MPTP-dependent degeneration process of dopaminergic neurons in the SNpc.

Expression levels of IPAS in the SNpc neurons of patients with sporadic PD were investigated. Formalin-fixed, paraffin-embedded sections of the midbrain of six patients and six neurologically normal control individuals were analyzed by immunohistochemistry, and IPAS expression levels were compared. The intracellular distribution of IPAS was similar to that of TH-positive neurons in MPTP-treated mice. The intensity of IPAS immunostaining was significantly greater in the neurons of sporadic PD patients than in those of control individuals. This result suggests that IPAS contributes to the neurodegeneration of dopaminergic neurons in the SNpc of patients with PD.

Although involvement of IPAS in apoptosis of dopaminergic neurons in PD was demonstrated, several questions remain to be addressed. First, an extensive search for alternative splice variants of HIF-3*α* is necessary that would allow elucidation of unknown variants using the apoptosis-related last exon. The variants may exert proapoptotic activity similar to IPAS in PD and/or other neurodegenerative diseases. Second, posttranslational modification of IPAS that regulates its proapoptotic activity needs to be resolved. In particular, analysis of IPAS phosphorylation by various kinases that exacerbates apoptosis in PD^10^ is needed. Last, involvement of IPAS in other neurodegenerative diseases needs to be investigated. Oxidative stress has an important role in delayed neuronal cell death following ischemic stroke. As IPAS is effectively induced by reactive oxygen species-generating intermittent hypoxia,^4^ it could be that IPAS is involved in the neuronal cell loss in cerebral infarction. It is also necessary to elucidate the role of IPAS in other neurodegenerative diseases, such as ALS and Alzheimer's disease, in which oxidative stress is postulated to be involved in their etiology.

## Figures and Tables

**Figure 1:**
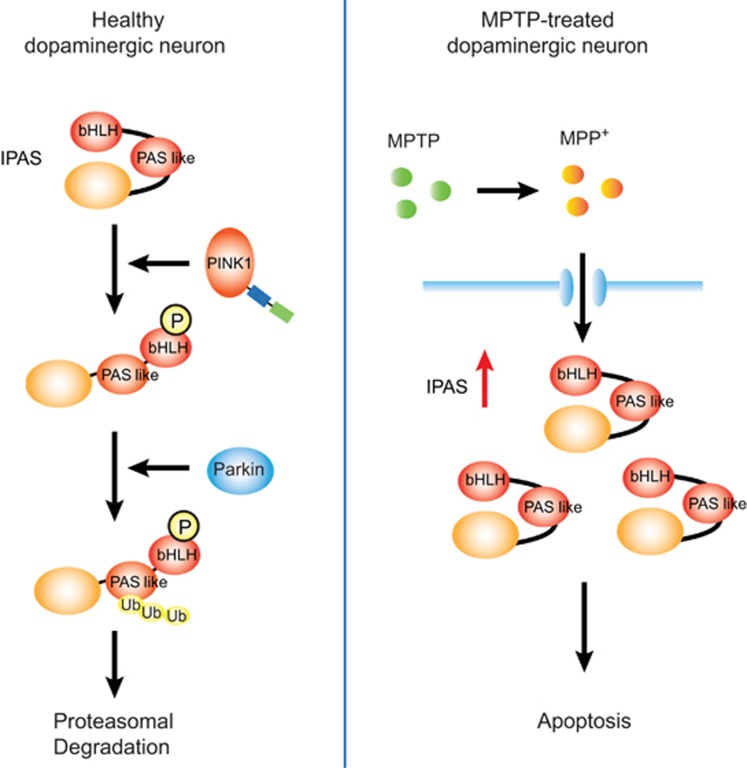
The PINK1-Parkin pathway regulates mitochondrial apoptosis by the breakdown of IPAS. In healthy neurons, IPAS Thr12 is phosphorylated by PINK1. This phosphorylation may induce conformational changes in IPAS to make the C-terminal region accessible to Parkin, and Parkin ubiquitinates IPAS for proteasomal degradation (left). IPAS is strongly induced in dopaminergic neurons of SNpc following administration of MPTP, and accumulating IPAS induces apoptosis in the neurons (right). bHLH, basic helix–loop–helix motif; PAS like, PAS-like domain; Ub, ubiquitin; MPP^+^, 1-methyl-4-phenylpyridinium
